# P. Thomas: I See Things Differently: A First Look at Autism: B.E.S Publishing, New York, 2014, 32 pp., ISBN 978-1-4380-0479-2, $6.79 (Paperback)

**DOI:** 10.1007/s10803-020-04378-7

**Published:** 2020-01-31

**Authors:** Kuvish

**Affiliations:** Southern Connecticut State University, 501 Crescent St, New Haven, CT 06515 USA

This book is intended for children who may have siblings or classmates with autism. Pat Thomas, the author of, I See Things Differently, strives to promote understanding of what autism is and what it feels like, from the perspective of the child with autism. Children hear all different things outside their home about what autism is and isn't. Parents and teachers have this major role in promoting an understanding of what autism is. They also could find age appropriate ways to encourage interaction among peers, such as helping with homework or walking home. This book includes sections called “what about you”, where children can talk and listen to allowing children to make connections from their experiences.

I See Things Differently starts with a brief description and followed through with common questions, misconceptions and includes information regarding emotions, behaviors, and who, typically, is diagnosed with autism. Speaking inclusively, the author suggests the reader may know someone with autism and elaborate on if its someone from school, a family member, or someone in their neighborhood. Even if they do not directly know someone with autism, the author provides a foundation explaining how the person with autism may seem ‘different’. “You may have seen them behaving in ways that seem strange to you, and wondered why they do these things(Thomas 2014)”, in the beginning of the book, this line helped build a community for readers to understand and acknowledge the base of autism, even without having to know someone with autism. The author wrote this book for readers make connections and understanding different perspectives. The author elaborates on ‘wearing the other person's shoes’ and how personal feelings, such as being worried, nervous, scared or different from people are common feelings, people with autism feel. Having empathy and understanding other perspectives is a hard task for anyone; people with autism may be unable to explain why they do certain things or may not be able to speak at all to explain why a situation occurred. After providing examples the author, wrote another brief introduction about autism. Thomas explained that autism affects the way the brain works, and discussed how it makes people behave or react differently. He discusses the way ‘normal’ people’s brains make sense of the world compared to people with autism and includes the first ‘what about you’ discussion, “do you know someone who has autism? Can you say what you think autism is?” These question/connection points in the book could be helpful to have children hear or tell their experiences. The author includes a description of what autism looks like, “They may look the same as everyone else on the outside, but inside they can feel like everyone around them is speaking a different language- or from a different planet! (Thomas 2014)”. By imputing examples of behaviors and emotions of people with autism, throughout the book, provides readers with a better grasp on autism. Behaviors, emotions and actions are all different depending on the person. Thomas provides readers with a few examples of what may cause people with autism to be stressed, upset, angry. These examples are big crowds, loud noises, repeating words and moving their bodies, slight changes and even having eye contact in a conversation that may cause behaviors or emotional instability, for those moments. This book identifies some habits that help people with autism feel safe and able to deal with their feelings. Pulling in the reader with another ‘what about you’ discussion, that allows people to describe and identify other habits they have encountered as well as, giving them time and guidance to help them understand the major keys with autism. Comparing how ‘normal’ people and people with autism handle things they don't want to see, hear or do. The author, Thomas, does a great job in comparing and explaining the common differences, as well as, how to understand and spot the differences in actions, behaviors, emotions, communications and other aspects of life. Thomas talks about how people with autism ‘aren't bad or behaving wrongly’ and how they're just ‘different’ and it’s a good thing to be different. Some people with autism have talents and skills such as being good at music, painting or remember specific things. Lastly, the author includes tips on how to be a good friend with patience and kindness, but no expectations, especially with “growing out of it”. Understanding autism and knowing they see, hear, feel, act differently and not limiting them to expectations is the perfect way to start and create relationships with people with autism.

Pat Thomas started broad and then continuously hit common questions and important introduction information about autism and people with autism. This was a great book to begin a classroom with a peer with autism or just information to open the students up to someone who may have autism in the school, community, and access to insightful information if a peer has a sibling with autism. This book is a great resource, that I would highly recommend to guide children into understanding autism in multiple perspectives that will provide knowledge to the readers and tips on forming a foundation of friendship with people with autism.
